# Effects of High Glucose on the Expression of LAMA1 and Biological Behavior of Choroid Retinal Endothelial Cells

**DOI:** 10.1155/2018/7504614

**Published:** 2018-06-05

**Authors:** Guangwei Song, Da Lin, Licheng Bao, Qi Jiang, Yinan Zhang, Haihua Zheng, Qianying Gao

**Affiliations:** ^1^The Second Affiliated Hospital and Yuying Children's Hospital of Wenzhou Medical University, Wenzhou 325027, China; ^2^The Second School of Medicine, Wenzhou Medical University, Wenzhou 325027, China

## Abstract

Hyperglycemia is one of the main causes of proliferative diabetic retinopathy (PDR) characterized by thickening of the vascular basement membrane. Laminin alpha 1 (LAMA1) is a primary component of laminin, a major protein constituent of the basement membrane. In this study, we investigated the role of LAMA1 in the development of PDR. Retinal choroidal vascular endothelial cells (RF/6A line) were exposed to glucose at different concentrations (5 mM, 15 mM, 25 mM, and 35 mM) and analyzed for cell growth, migration, proliferation, and adhesion. LAMA1 expression was examined 24 and 48 h following glucose treatment using Western blotting, RT-PCR, and immunofluorescence. The results showed that the proliferation, migration, and adhesion of RF/6A cells were increased by high glucose, whereas LAMA1 expression was slightly higher at 15 mM but decreased at 25 mM and 35 mM glucose compared to control. Thus, the changes in the biological behavior of high glucose-exposed retinal vascular endothelial cells correspond to variations in LAMA1 expression, indicating a possibility for LAMA1 involvement in PDR development. Our findings suggest that LAMA1 may play a role in PDR and, thus, may serve as a potential target for DR diagnosis and/or treatment.

## 1. Introduction

Diabetic retinopathy (DR) is one of the major microvascular complications caused by long-term hyperglycemia, and is also one of the most serious causes of blindness worldwide [1]. It is estimated that about one third of diabetic patients can develop DR [2] as a result of long-term accumulation of damaged small retinal vessels due to chronically poor blood glucose control [3]. Hyperglycemia is associated with the formation of advanced glycation end products, oxidative stress, inflammation, and neovascularization. These processes induce changes in the microvascular system, such as increased synthesis of extracellular matrix (ECM) proteins and thickening of the capillary basement membrane, which are the main pathological features of diabetic microangiopathy [4, 5].

DR is characterized by endothelial cell dysfunction, basement membrane thickening, increased fibrogenesis, and retinal detachment caused by fiber membrane retraction; in the advanced or proliferative (PDR) stage, blood vessels proliferate, resulting in neovascularization [6]. The thickened basement membrane is considered to be the consequence of upregulated expression of vascular basement membrane components such as type IV collagen, fibronectin, and laminin [5]. Oxidative damage is an important factor in endothelial cell dysfunction, and high glucose can cause excessive generation of reactive oxygen species (ROS) and activation of the protein kinase C (PKC)-beta pathway, leading to increased expression of ECM proteins and vascular endothelial growth factor (VEGF), and changes in endothelial cell permeability [7]. The ECM provides a scaffold required for vascular endothelial cells to form blood vessels, whereas adhesion to specific ECM components via integrins triggers intracellular signaling critically required for endothelial cell proliferation, migration, survival, and tube morphogenesis [8]. Thus, changes in the ECM are involved in endothelial dysfunction, basement membrane thickening, and neovascularization.

Laminin is one of the major ECM components participating in basement membrane formation. By binding to cell surface receptors, laminin induces different signal transduction pathways, which influence cell behavior such as adhesion, differentiation, migration, phenotype stability, and resistance to anoikis [9]. A previous study has shown that serum laminin can be used as a biomarker of DR associated with vascular endothelial dysfunction [10]. Laminin is a trimeric glycoprotein consisting of three chains, alpha, beta, and gamma and has been found in at least 16 different isoforms [11]. It was reported that the expression of laminin chains is regulated both spatially and temporally [12], suggesting that different laminin isoforms have distinct functional roles. A number of laminin isoforms are expressed in the basement membrane of retinal blood vessels and where they could influence vascular development [13].

Laminin *α* 1 (LAMA1), the most conserved subunit in all laminin isoforms, plays an essential role in embryonic development and promotes neurite outgrowth [14]. In mice, LAMA1 was shown to be expressed in the internal limiting membrane (ILM) between the retina and the vitreous body and in Bruch's membrane of the choroid, and it was reported that LAMA1 deficiency could cause abnormal cell adhesion and migration, leading to altered retinal angiogenesis, persistent vitreal fibroplasias, epiretinal membrane formation, and peripheral retinal degeneration [15, 16]. Epiretinal membranes were described in both of these mutants (LAMA1 knockout mice) which had characteristics similar to human persistent fetal vasculature (PFV) and proliferative vitreoretinopathy (PVR).

The aim of this study was to examine the role of LAMA1 in the development of PDR. As vascular endothelial cells play an important role in DR development [6], we used retinal choroidal vascular endothelial cells, which were exposed to different glucose concentrations and analyzed for cell behavior and LAMA1 expression.

## 2. Materials and Methods

### 2.1. Reagents and Antibodies

Rabbit anti-human LAMA1 antibody (sc-5582) was from Santa Cruz Biotechnology (Dallas, TX, USA). Mouse anti-human GAPDH antibody, goat anti-rabbit IgG H&L (ab150077), and goat anti-mouse IgG H&L were purchased from Abcam (Cambridge, MA, USA). TRIzol reagent and DAPI (4′,6-diamidino-2-phenylindole) were obtained from Invitrogen (Carlsbad, CA, USA). The PrimeScript RT reagent kit with gDNA Eraser and SYBR Premix Ex Taq™ II (Tli RNaseH Plus) kit were from Takara Clontech (Kyoto, Japan). Cell Counting Kit-8 (CCK-8) was from Dojindo (Kumamoto, Japan), and 4-well culture inserts were from Ibidi (cat. number 80469; Martinsried, Germany).

### 2.2. Cell Culture

Rhesus choroid retinal endothelial cells (RF/6A line; Chi Scientific, Jiangsu, China) were cultured in DMEM (Gibco, Life Technologies, NY, USA) supplemented with 10% fetal bovine serum (FBS; Gibco) and 1% penicillin/streptomycin (Gibco) at 37°C in a humidified incubator with 95% air-5% CO_2_. Cells reaching at least 80% confluence were lifted by treatment with 0.25% trypsin/0.02% EDTA and passaged. To model hyperglycemic conditions, cells were grown in medium supplemented with 15 mmol/L, 25 mmol/L, and 35 mmol/L glucose for different times; control cells were grown at 5 mmol/L glucose.

### 2.3. Western Blotting

LAMA1 protein expression was assessed by Western blotting. RF/6A cells were washed twice with PBS and lysed with cold radioimmunoprecipitation assay (RIPA) buffer containing protease inhibitor phenylmethylsulfonyl fluoride (PMSF; Shanghai Beyotime Biotech Co., China). Lysed cells were collected using a cell scraper, transferred to Eppendorf tubes, and centrifuged for 30 min at 12,000 rpm (4°C) to obtain supernatants. A BCA protein assay kit (Shanghai Beyotime Biotech Co.) was used to determine protein concentrations. Protein samples (20 *μ*g protein/well) were subjected to sodium dodecyl sulfate polyacrylamide gel electrophoresis (SDS-PAGE) and transferred to polyvinylidene difluoride (PVDF) membranes, which were blocked in 5% BSA for 2 h at room temperature. After that, the membranes were incubated with antibodies to LAMA1 (1 : 1000) overnight, washed three times with Tris-buffered saline containing 0.05% Tween 20 for 30 min each, and incubated with HRP-conjugated secondary antibodies for 2 h at room temperature. Specific bands were visualized using Immobilon Western Chemiluminescent HRP Substrate reagents (Millipore Corporation, MA, USA) in a transilluminator (ChemiDoc; Bio-Rad, Hercules, CA, USA). Band densities were quantified using the Image-Pro Plus software.

### 2.4. Real-Time PCR

LAMA1 mRNA expression in glucose-treated RF/6A cells was detected by real-time PCR. Total RNA was extracted using TRIzol according to the manufacturer's instructions, and its concentration was assessed by spectrophotometry; an A_260_/_280_ ratio of 1.8–2.0 was considered acceptable. cDNA was synthesized using the PrimeScript RT reagent kit with gDNA Eraser according to the manufacturer's protocol and used as a template for PCR amplification. LAMA1 primers were synthesized by Sangon Corporation (Shanghai, China). Real-time PCR (SYBR Green) was performed according to the manufacturer's instructions in a LightCycler 480 system (Roche, Basel, Switzerland) in a volume of 20 *μ*L containing SYBR Premix Ex Taq II (Takara RR820A), cDNA, and specific primers. Relative mRNA expression of LAMA1 was quantified by the comparative 2^−∆∆ct^ method after normalization to that of GAPDH. Primer sequences are shown in Table 1.

### 2.5. Immunofluorescence Staining

RF/6A cells were seeded onto glass coverslips, incubated at 37°C and 5% CO_2_ until 80% confluence, fixed with 4% paraformaldehyde for 15 min at room temperature, washed three times with PBS, permeabilized with 0.1% Triton X-100 for 4 min, blocked in 3% BSA for 1 h, and incubated with anti-LAMA1 antibodies (1 : 50) overnight at 4°C. Cells were washed three times in PBS and incubated with goat anti-rabbit secondary antibodies for 1 h at 37°C followed by DAPI staining for 10 min to visualize nuclei. Fluorescence images were obtained under a fluorescence microscope (model DM6000; Leica, Wetzlar, Germany).

### 2.6. Cell Growth Assay

RF/6A cells were cultured in normal glucose medium (5 mM) for 24 h to allow cell adherence; then, the medium was removed, and cell monolayers were washed once with PBS and incubated in medium supplemented with different concentrations of glucose. The bottom was marked to determine the position of observed cells; the growth of cells at the same position was observed at different time points (0, 1, 2, 3, and 4 days) under a microscope (Axio Observer A1; Carl Zeiss, Oberkochen, Germany).

### 2.7. Cell Proliferation Assay

Cell proliferation was measured using the CCK-8 method in accordance with the manufacturer's protocol. Briefly, RF/6A cells (7 × 10^3^ cells/100 *μ*L/well) were seeded into 96-well plates overnight and then cultured at different glucose concentrations for 96 h; normal glucose (5 mM) served as control. Cell proliferation was assessed every 24 h after addition of 10 *μ*L of the CCK-8 solution for 90 min at 37°C and subsequent measuring of absorbance at 450 nm using a microplate reader (Synergy H1; BioTek, Winooski, VT, USA). The optical density reflected the proliferation of RF/6A cells. Each experiment was performed in five replicates, and detection was repeated three times per group to ensure accuracy of measurements. The experiment was repeated independently three times, and the mean values obtained in each experiment were used for statistical analysis.

### 2.8. Wound Healing Assay

Cell migration ability was assessed using wound healing assay by 4 Well Insert (Ibidi cat. number 80469). The Culture-Insert 4 Well consists of four wells, representing the four quarters of the round Culture-Insert. The wells are separated by a wall of 500 *μ*m. When the wells are filled with adherent cells, a cell-free gap of approx. 500 *μ*m is created between the adjacent wells after removing the Culture–Insert 4 Well. RF/6A cells (4 × 10^5^ cells/mL) were plated into the four wells of a culture insert located at the center of a 35 mm culture dish. After 24 h, when the cells reached 90% confluence, the culture insert was removed to reveal the wound gap, and micrographs (baseline time 0) were taken at ×10 magnification using a digital camera attached to an inverted microscope (Axio Observer A1). Then, cells were washed with PBS to remove floating cells, fresh medium containing different glucose concentrations (5 mM, 15 mM, 25 mM, or 35 mM) was added, and cells were cultured for 72 h. Wound healing was monitored by taking micrographs at the same coordinates every 24 h. Quantitative analysis was performed by measuring the gap size of the wound using the Image-Pro Plus software and the results were presented as the percentage of the wound coverage; complete coverage was defined as 100%. Experiments were repeated three times.

### 2.9. Cell Adhesion Assay

Cell adhesion ability was measured using the modified CCK-8 method. Cell suspension (7 × 10^4^ cells/mL) was prepared in medium containing different concentrations of glucose, and 100 *μ*L of cell suspension was placed into each well of 96-well plates, which were then placed in 37°C incubator for 2, 4, 6, and 8 h. Then, cell monolayers were washed twice with PBS to remove unattached cells, and 100 *μ*L of fresh culture medium containing 10 *μ*L CCK-8 solution was added for 2 h. The assay was performed with five replicates. The absorbance was measured in a microplate reader; higher absorbance values reflected higher number of adhered cells.

### 2.10. Statistical Analysis

Each experiment was repeated at least three times and the data are presented as the mean ± standard deviation (SD). To assess the significance of differences between groups in multiple comparisons, one-way ANOVA was used; two-sided *P* values less than <0.05 indicated statistical significance. All statistical analyses were performed using the Prism 6.0 software (GraphPad Software, San Diego, CA, USA).

## 3. Results

### 3.1. Effects of High Glucose on the Growth of RF/6A Cells

**Figure 1:**
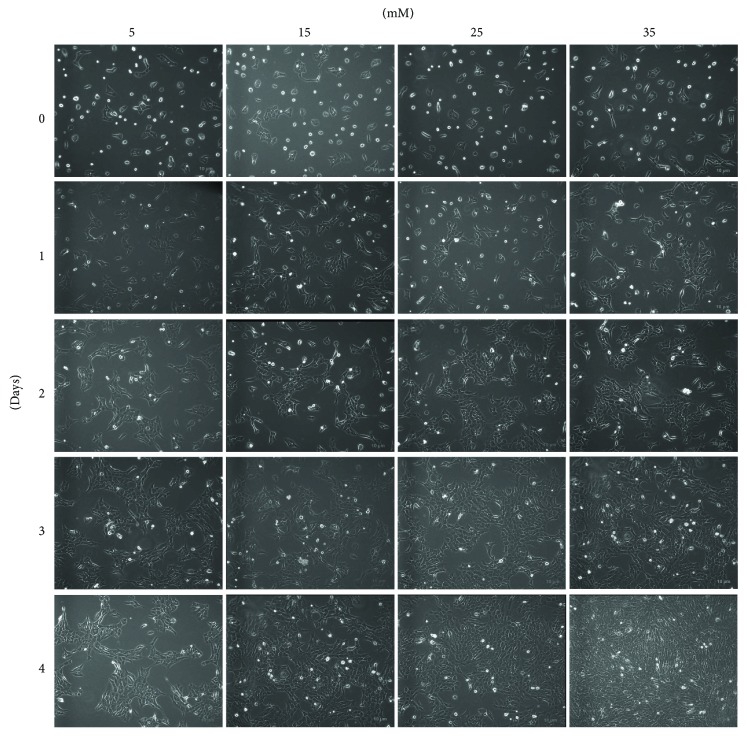
Effects of high glucose on the growth of RF/6A cells. RF/6A cells were grown in medium supplemented with different concentrations of glucose for the indicated times.

RF/6A cells were cultured with different glucose concentrations and their growth was analyzed at 1, 2, 3, and 4 days after treatment. After 1 day of culture, no significant difference in cell growth was observed compared with control (normal glucose concentration, 5 mM); however, further culture in hyperglycemic conditions induced cell growth in a glucose concentration-dependent manner (Figure 1).

### 3.2. Effects of High Glucose on LAMA1 Expression in RF/6A Cells

#### 3.2.1. Localization of LAMA1 Expression

**Figure 2:**
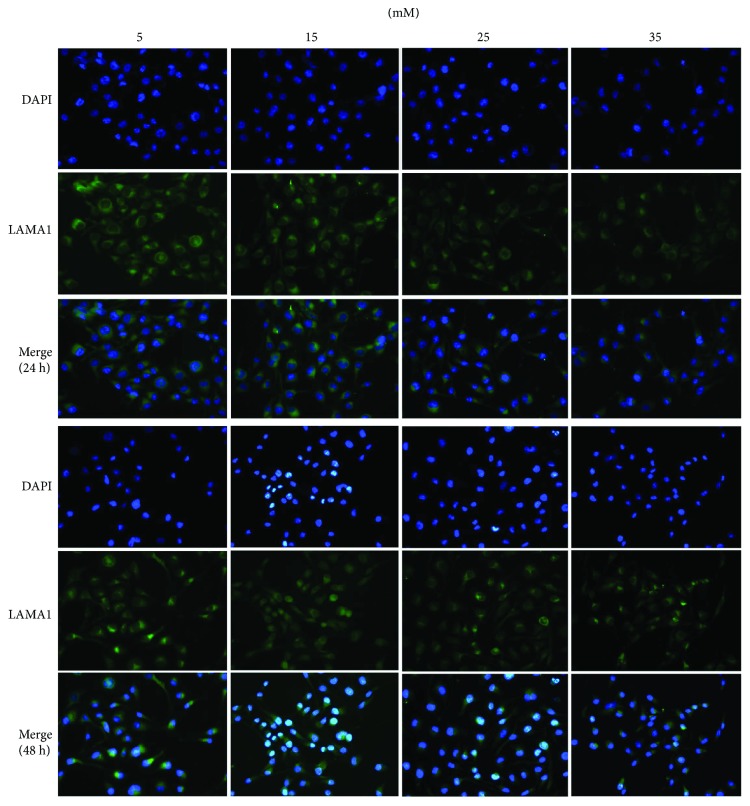
Immunocytochemistry analysis of LAMA1 expression in RF/6A cells treated with different concentrations of glucose for 24 and 48 h.

Immunocytochemistry analysis indicated that LAMA1 was expressed mainly in the cytoplasm. LAMA1 expression was not affected by the treatment with 15 mM glucose for 24 h and 48 h, but was reduced by that with 25 mM and 35 mM glucose compared with control (5 mM) (Figure 2).

#### 3.2.2. LAMA1 Protein Expression

**Figure 3:**
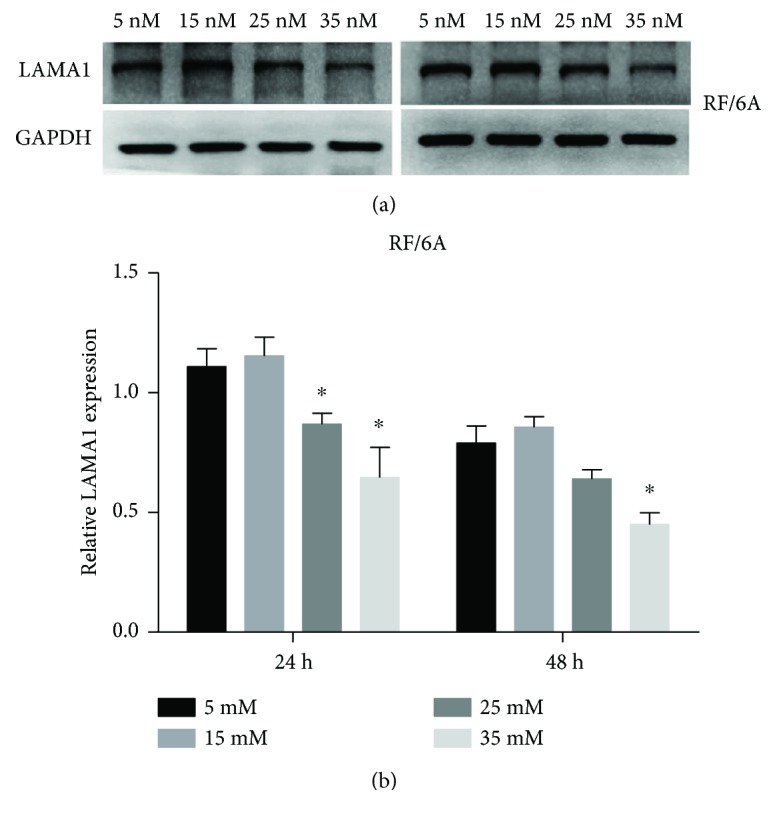
Expression of LAMA1 protein in RF/6A cells. RF/6A cells were treated with different concentrations of glucose for 24 and 48 h and LAMA1 expression was analyzed by Western blotting; GAPDH was used as loading control. (a) Representative gel images. Left, 24 h; right, 48 h. (b) Quantitative analysis of LAMA1 expression performed using the ImageJ software. The data are presented as the mean ± SD of three independent experiments; ^∗^*P* < 0.05.

The expression of LAMA1 protein was examined by Western blotting, which showed that 15 mM glucose slightly induced LAMA1 expression, but the difference with control (5 mM) was not statistically significant (*P* > 0.05). However, the increase in glucose concentration significantly downregulated LAMA1 protein expression, especially in the 35 mM group (*P* < 0.05), and the effect was more pronounced after 48 h exposure to high glucose (Figure 3).

#### 3.2.3. LAMA1 mRNA Expression

**Figure 4:**
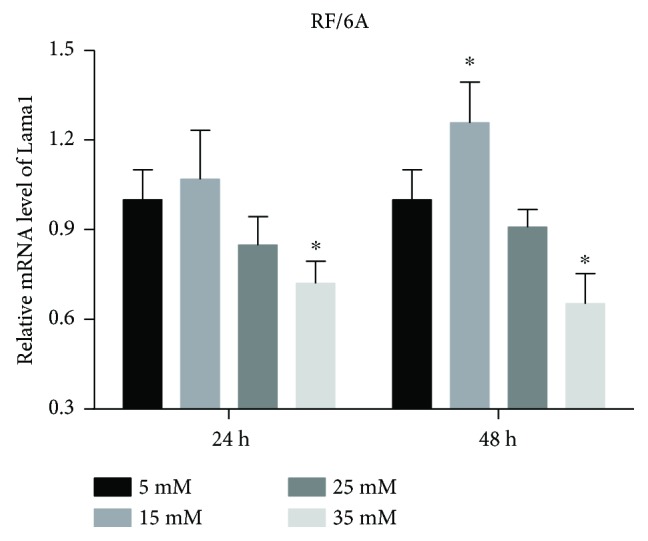
LAMA1 mRNA expression. RF/6A cells were treated with different concentrations of glucose for 24 and 48 h and analyzed for LAMA1 mRNA levels by real-time PCR. There was a significant decrease of LAMA1 mRNA expression in cells exposed to 35 mM glucose both at 24 and 48 h. The data are presented as the mean ± SD of three independent experiments; ^∗^*P* < 0.05.

Real-time PCR analysis of LAMA1 transcription in RF/6A cells showed that the exposure to 35 mM glucose significantly decreased LAMA1 mRNA levels compared to control (5 mM) (*P* < 0.05). There was also a slight decrease by 25 mM glucose, but the difference with control did not reach statistical significance (*P* > 0.05). These results are consistent with LAMA1 protein expression detected by Western blotting (Figure 4).

### 3.3. Effects of High Glucose on the Migration Ability of RF/6A Cells

**Figure 5:**
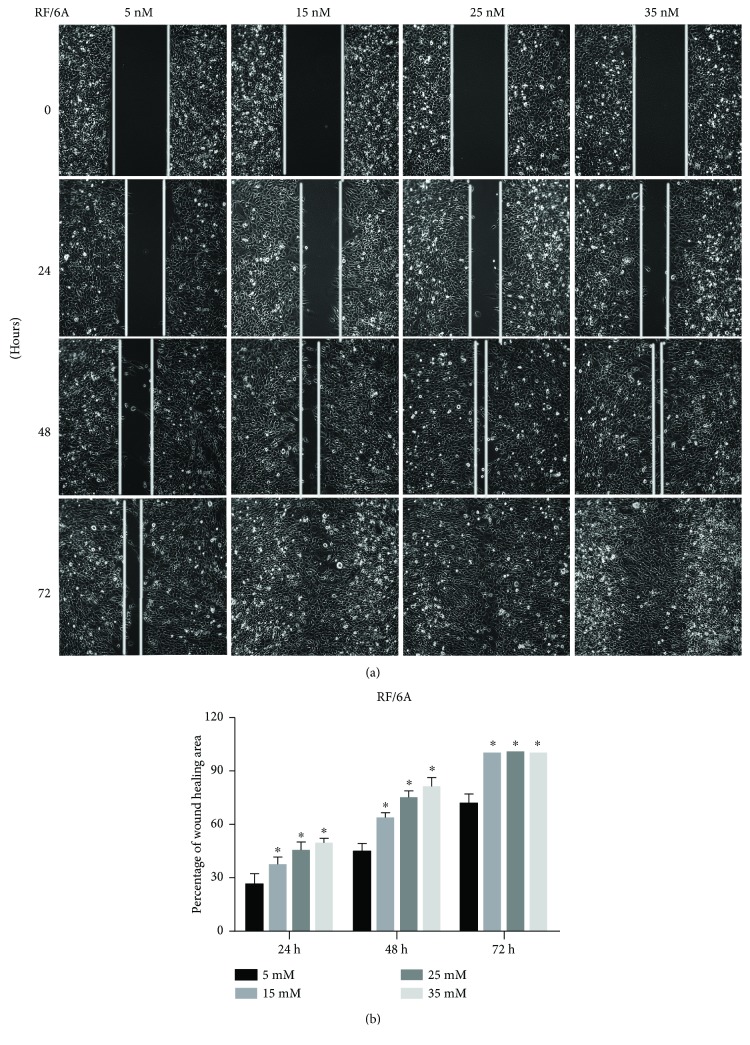
Cell migration ability. RF/6A cells grown at different concentrations of glucose for 24, 48, and 72 h were analyzed by the wound healing assay. (a) Representative microscopic images of wound healing. (b) Quantitative analysis of the gap size measured using the Image-Pro Plus software; ^∗^*P* < 0.05.

The effect of high glucose concentration on retinal endothelial cell migration ability was analyzed by the wound healing assay. The results showed that wound healing was accelerated by high glucose in a concentration-dependent manner, indicating the induction of cell migration ability. At 24 h, the healed area was increased to 37.12 ± 4.83% (15 mM), 45.52 ± 4.37% (25 mM), and 49.77 ± 2.35% (35 mM) compared to 26.4 ± 5.91% in the control group (5 mM), and the difference was statistically significant (*P* < 0.05). At 48 h, the healed area was increased to 63.42 ± 2.98% (15 mM), 75.01 ± 3.49% (25 mM), and 80.79 ± 5.53% (35 mM) compared to 44.96 ± 4.56% in the control group (5 mM), and the difference was statistically significant (*P* < 0.05). After 72 h, the healed area in the control group was 71.44 ± 5.29, which was significantly lower (*P* < 0.05) than that in high glucose groups (100%) (Figure 5).

### 3.4. Effects of High Glucose on the Proliferation of RF/6A Cells

**Figure 6:**
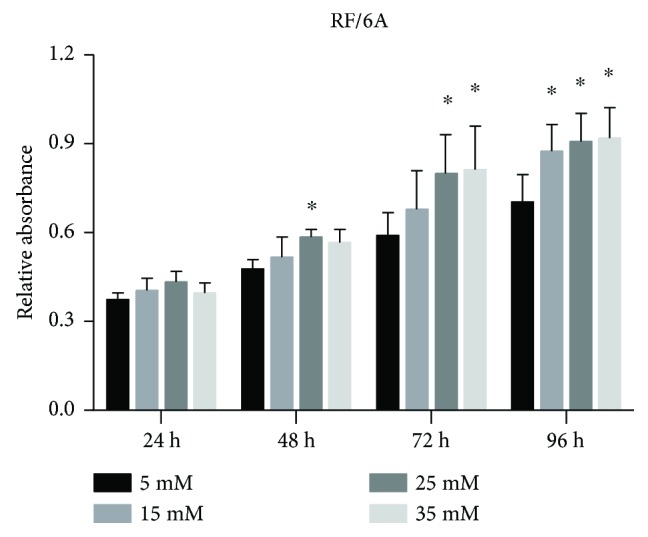
Proliferation of RF/6A cells exposed to high glucose concentrations. RF/6A cells grown at different concentrations of glucose for 96 h were analyzed for cell proliferation every 24 h using the CCK-8 assay. The data are presented as the mean ± SD of three independent experiments; ^∗^*P* < 0.05.

The CCK-8 assay showed that RF/6A cell proliferation in high glucose-supplemented medium was induced compared to control (5 mM), which was consistent with microscopic observations of cell growth. At 24 h, there was no statistically significant difference between the groups. However, at 48 h, an increase in cell proliferation compared to control was detected in the 25 mM group (*P* < 0.05), whereas at 72 h, it was revealed in the 25 mM and 35 mM groups (*P* < 0.05), and at 96 h, it was observed in all cells exposed to high glucose (*P* < 0.05) (Figure 6).

### 3.5. Effects of High Glucose on the Adhesion Ability of RF/6A Cells

**Figure 7:**
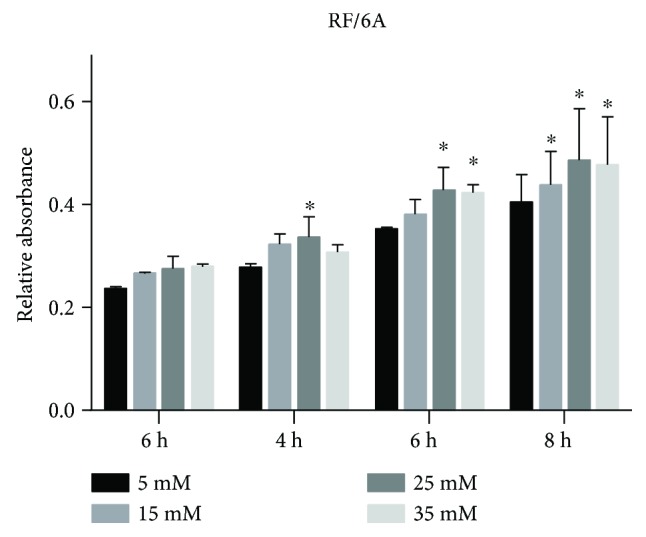
Adhesion ability of RF/6A cells exposed to high glucose. RF/6A cells suspended in medium containing different concentrations of glucose were seeded in 96-well plates and analyzed for adhesion after indicated times using the modified CCK-8 method. The data are presented as the mean ± SD of three independent experiments; ^∗^*P* < 0.05.

The adhesion of cells cultured at the elevated glucose concentrations showed a trend for increase compared to control, and the difference between control and 25 mM and 35 mM glucose-treated cells was statistically significant at 6 and 8 h (*P* < 0.05; Figure 7).

## 4. Discussion

DR is one of the leading complications of diabetes worldwide and the primary cause of blindness in developed countries [17]. DR incidence is expected to grow in the next few years, and the disease is predicted to affect nearly 600 million people by the year 2035 [18]. However, the exact pathogenesis of DR is still unclear and is considered to be induced through several mechanisms: (1) polyol pathway, (2) advanced glycation end products, (3) PKC activation, (4) genetic factors, (5) inflammation, and (6) oxidative stress [6, 19]. Each mechanism can cause endothelial cell dysfunction, resulting in a series of physiological and biochemical abnormalities such as retinal ischemia, vascular permeability change, macular edema, VEGF upregulation, and neovascularization, which eventually lead to PDR [20]. Therefore, it is important to study the reaction of retinal endothelial cells to hyperglycemic condition characteristic for diabetes.

In recent years, research on PDR has been focused on the role of growth factors, especially VEGF-A, in ocular angiogenesis [21, 22], whereas less attention has been paid to the ECM, which is important for the development of retinal vessels and pathological neovascularization [8, 23]. Endothelial cells secrete proteases that degrade the original vascular basement membrane and cause its components to diffuse from the blood vessels to the surrounding ECM [24]. Laminin is one of the important ECM components that, together with collagen, form the basement membrane. In 1992, Ingber et al. [25] found that interactions between endothelial cells through basement membrane components play a critical role in angiogenesis. Among ECM proteins, LAMA1 is highly expressed in the eye structures, especially in retinal vessels and the lens [26]. Interestingly, several studies indicate that high glucose can induce the secretion of ECM proteins (collagen IV, fibronectin, and laminin) through signaling pathways such as TGF-*β*/Smad and PI3K/AKT [27–29]; however, our results showed that at the increase of glucose concentration, LAMA1 expression was only slightly upregulated and then showed a marked downward trend. Ning et al. [30] found that LAMA1 plays a critical role in the function and aging of the kidneys by regulating the mesangial cell population and mesangial matrix deposition through TGF-*β*/Smad signaling. TGF-*β* is a secreted signaling molecule with a fibrogenic effect that regulates diverse cellular processes, including proliferation, differentiation, migration, and apoptosis, and is being recognized as an important contributor to DR pathogenesis [31]. As in vitro and in vivo studies indicate that LAMA1 deficiency promotes mesangial cell proliferation and that this pathological change is very similar to diabetic nephropathy [30], we hypothesize that hyperglycemia can influence the proliferation of retinal endothelial cells through the LAMA1-TGF-*β* axis. LAMA1 expression could be first increased by high glucose through a compensatory mechanism and then start to decline, which would negatively regulate TGF-*β* signaling, inducing cell proliferation and migration. However, our present findings cannot confirm this hypothesis, which should be tested in further studies. The decrease of LAMA1 expression in RF/6A cells under high glucose conditions suggests that LAMA1 may serve as a protective factor in DR. Studies in mice indicate that mutations in the Lama1 gene promoted the formation of vitreoretinal blood vessels and the epiretinal membrane, supported persistence of fetal vasculature, and could cause vitreal fibroplasia and abnormal vascular development [15, 32]. Taken together, these and our data indicate that LAMA1 deficiency plays a role in DR pathogenesis.

Our study shows that high glucose promotes the growth and proliferation of endothelial cells and accelerates their migration, providing a material basis for angiogenesis and fiber proliferation. These results are consistent with a report by Beltramo et al. [33] that in the early stages of DR, hyperglycemia can cause a loss of pericytes, reducing their association with endothelial cells and increasing vascular permeability and endothelial cell proliferation, which ultimately leads to neovascularization. In contrast, other studies showed that high glucose inhibited endothelial cell proliferation and promoted apoptosis, causing endothelial damage, which results in diabetes-related vascular complications [34, 35]. However, it is well known that hyperglycemia is the main cause of PDR characterized by pathological neovascularization due to cell proliferation. Therefore, diverse effects of high glucose on endothelial cells suggest the existence of multiple molecular mechanisms underlying the development of PDR.

In summary, our study indicates that glucose at high concentration downregulates the expression of LAMA1, which corresponds to increased adhesion, proliferation, and migration of choroid retinal endothelial cells, suggesting that LAMA1 may exert a protective effect against DR. Thus, LAMA1 may be used as a new target for diagnosis and/or treatment of DR, providing a new direction for DR clinical research. Further studies are required to address specific molecular mechanisms and signaling pathways underlying LAMA1 role in DR development.

## Figures and Tables

**Table 1:** Primer sequences used for PCR.

Gene	Sequence	Length
LAMA1 (forward)	5′- GTT TCG AAC CTC CTC GCA GA-3′	88 bp
LAMA1 (reverse)	5′- CTT GCC GTC CAC AAG CTC TAG T-3′	
GAPDH (forward)	5′- GAT TCC ACC CAT GGC AAA TT-3′	103 bp
GAPDH (reverse)	5′- TCT CGC TCC TGG AAG ATG GT-3′	

## Data Availability

The data used to support the findings of this study are available from the corresponding author upon request.
